# Methylene Blue Dye Adsorption from Wastewater Using Hydroxyapatite/Gold Nanocomposite: Kinetic and Thermodynamics Studies

**DOI:** 10.3390/nano11061403

**Published:** 2021-05-26

**Authors:** Kashma Sharma, Shreya Sharma, Vipasha Sharma, Pawan Kumar Mishra, Adam Ekielski, Vishal Sharma, Vijay Kumar

**Affiliations:** 1 Institute of Forensic Science & Criminology, Panjab University, Chandigarh 160014, India; shama2788@gmail.com (K.S.); sharmashreya.m1223@gmail.com (S.S.); 2 Department of Chemistry, DAV College, Sector-10, Chandigarh 160011, India; 3 Department of Biotechnology, Chandigarh University, Gharuan 140413, India; sharma.vipasha@gmail.com; 4 Faculty of Business and Economics, Mendel University in Brno, 61300 Brno, Czech Republic; xmishra2@gmail.com; 5 Department of Production Engineering, Warsaw University of Life Sciences, 02-776 Warsaw, Poland; adam_ekielski@sggw.edu.pl; 6 Department of Physics, National Institute of Technology, Srinagar 190006, India

**Keywords:** hydroxyapatite, gold nanoparticles, nanocomposites, dye adsorption, dye adsorbed waste, antibacterial

## Abstract

The present work demonstrates the development of hydroxyapatite (HA)/gold (Au) nanocomposites to increase the adsorption of methylene blue (MB) dye from the wastewater. HA nanopowder was prepared via a wet chemical precipitation method by means of Ca(OH)_2_ and H_3_PO_4_ as starting materials. The biosynthesis of gold nanoparticles (AuNPs) has been reported for the first time by using the plant extract of *Acrocarpus fraxinifolius*. Finally, the as-prepared HA nanopowder was mixed with an optimized AuNPs solution to produce HA/Au nanocomposite. The prepared HA/Au nanocomposite was studied by using Fourier transform infrared spectroscopy (FTIR), X-ray diffraction (XRD), and Scanning Electron Microscopy (SEM) with Energy Dispersive X-Ray Analysis (EDX) analysis. Adsorption studies were executed by batch experiments on the synthesized composite. The effect of the amount of adsorbent, pH, dye concentration and temperature was studied. Pseudo-first-order and pseudo-second-order models were used to fit the kinetic data and the kinetic modeling results reflected that the experimental data is perfectly matched with the pseudo-first-order kinetic model. The dye adsorbed waste materials have also been investigated against *Pseudomonas aeruginosa*, *Micrococcus luteus*, and *Staphylococcus aureus* bacteria by the agar well diffusion method. The inhibition zones of dye adsorbed samples are more or less the same as compared to as-prepared samples. The results so obtained indicates the suitability of the synthesized sample to be exploited as an adsorbent for effective treatment of MB dye from wastewater and dye adsorbed waste as an effective antibacterial agent from an economic point of view.

## 1. Introduction

Water, by virtue of being a universal solvent, experiences contamination by a huge number of toxic compounds, comprising heavy metal ions, pesticides, fertilizers, pharmaceutical products, dyes, organic solvents, and so forth [1,2,3]. These substances, even at extraordinarily minute quantities, can motivate noteworthy contagion, thus crushing biological system elements [4,5,6,7]. Direct release of these toxic compounds from some of the sources into the local surroundings without any proper pre-treatment is taken into consideration as the primary menace to water safety [8]. The dye industry is recognized as the tenth most contaminating industry to the Water Rivers, as 17%–20% of industrial water waste is mainly because of the textile and dye industry [8,9]. Because of their abundant use, around 5000–10,000 tons of dye are thrown out into the waterways every year [8,10,11].

Methylene blue (MB) dye is a cationic dye that has potential use in the industry, such as in dyeing cotton, silk and wool [12,13,14]. In some cases, it may lead to eye burn, which results in long-lasting eye damage in humans and animals [12,15,16,17]. It may also cause short durations of fast heartbeats or shallow breathing in the case of inhalation [12]. Therefore, an effective treatment technology of any wastewater holding MB dye molecules are required to lower their harmful impacts on aquatic life and human health. Consequently, several decontamination technologies viz. microbiological degradation [18,19,20], advanced oxidation [20,21,22], adsorption [10,20], electrochemical techniques [23], coagulation and flocculation [20], ion exchange [24], reverse osmosis [25], membrane filtrations [20,26], and so forth have been developed over the years to provide clean water to humans, especially for drinking and daily household purposes. Among the various reported techniques, adsorption technology is a very prominent technique owing to its high efficacy, cost-effectiveness and ease [20]. Until now, large amounts of an adsorbent, such as hydrogels, polymers, clays, metal nanoparticles, and so forth, in raw form or with the modified surface, have been investigated for the abstraction of toxic compounds from wastewater [27,28,29,30,31]. Thus, there is a strong scientific motive to search for fairly efficient, cost-effective and easily accessible adsorbents for the removal of toxic compounds.

In this work, we explored the development of hydroxyapatite (HA)/gold (Au) nanocomposites for the rapid adsorption of a ubiquitous industrial pollutant MB dye. Among various organic and inorganic NPs, HA is viewed as one of the inorganic bio-ceramics [29]. It is considered a prospective material for various applications in a variety of fields [29,32,33]. It has certain properties such as biocompatibility, osteoconductivity, biodegradability and synthetic nanostructures, as well as an affinity for other materials [29]. There are many reports on HA and their hybrid or nanocomposites with various organic and inorganic materials for the elimination of various toxic compounds from aqueous media [29,34]. The elimination of lead ions by using nano HA synthesized from phosphogypsum waste was reported and they found the greatest adsorption was 769.23 mg/g for Pb ions [35]. Feng et al. [36] studied the adsorption behavior of magnetic nano HA for the discharge of Cd^2+^ and Zn^2+^ from aqueous media. Zhu et al. [37] focused on the cost-effective adsorption properties of a fluor-HA based composite for removing Cd^2+^ from the wastewater. Researchers have also been using HA/graphene hybrid nanocomposite for the successful abstraction of MB dye from wastewater [38]. The experimental data was in line with the pseudo-second-order kinetic model and the adsorption capacity of MB by hybrid nanocomposite enhanced with the initial MB concentration. The adsorption capacity was evaluated to be 333.3 mg/g for the studied system. Adeogun et al. [39] concentrated on the adsorption behavior of the bio-waste-derived HA composite for removing yellow 4 dye from aqueous media. Guan et al. [40] studied the Congo red dye adsorption of nanosized rod-like HA particles and concluded that the synthesized material could be a capable and green option for the current handling of industrial wastewater. Varaprasad et al. [29] synthesized nano HA-based carboxymethyl cellulose/acrylamide (CMC-AM) hydrogels and investigated the adsorption process of Acid Blue 113 dye.

The current work discusses the synthesis and characterization of HA/Au nanocomposite, which rapidly adsorbs cationic dye MB from its aqueous solution. Importantly, we also performed reusability experiments of dye adsorbed waste material to reduce the secondary waste and cost in antibacterial application. To the best of our knowledge, the plant extract of *Acrocarpus fraxinifolius* has not been used so far for the biosynthesis of AuNPs and their nanocomposite with HA nanopowder. In addition to this, the reaction mechanism and the limits of the thermodynamic studies were also investigated. The antibacterial activity of as-prepared and dye adsorbed nanocomposite samples have also been investigated against three bacterial, that is, *Pseudomonas aeruginosa*, *Micrococcus luteus* and *Staphylococcus aureus*.

## 2. Materials and Methods

### 2.1. Materials

CaO powder, H_3_PO_4_ and NH_3_ solution was procured from Sisco Research Laboratories Pvt. Ltd., Mumbai (India), chloroauric acid (HAuCl_4_) was procured from Loba Chemie Pvt. Ltd., Mumbai (India). Methylene blue (MB) dye, HCl and NaOH pellets were procured from Merck, Mumbai (India).

### 2.2. Preparation of Plant Extract

**Scheme 1:**
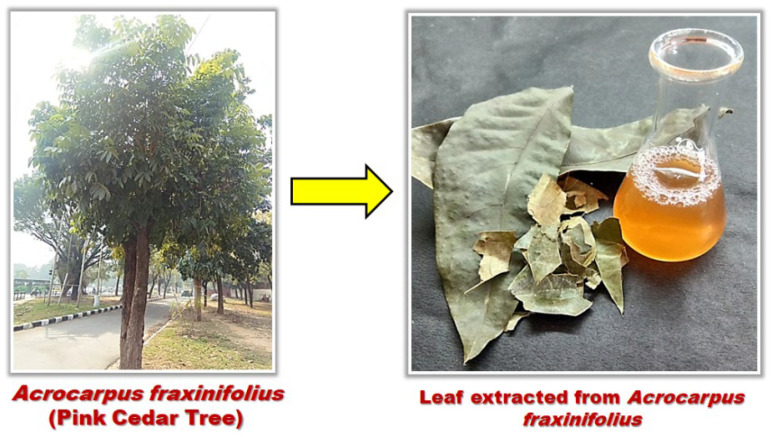
Preparation of plant extract.

The leaves of *Acrocarpus fraxinifolius* trees were collected and washed meticulously under tap water to clear out dirt and other trapped debris. Subsequently, the washed leaves were dried at an ambient temperature. Five grams of dried leaves were measured using analytical weighing balance and boiled in 200 mL of distilled water in a microwave for about 5 min of duration. Then it was gravity filtered using Whatman Filter paper (125 mm) to get a clear pale yellow colored aqueous extract of *Acrocarpus fraxinifolius* (Scheme 1). This extract was then centrifuged at 6000 rpm for 10 min and used for the further processes in this study.

### 2.3. Biosynthesis of Gold Nanoparticles (AuNPs)

**Scheme 2:**
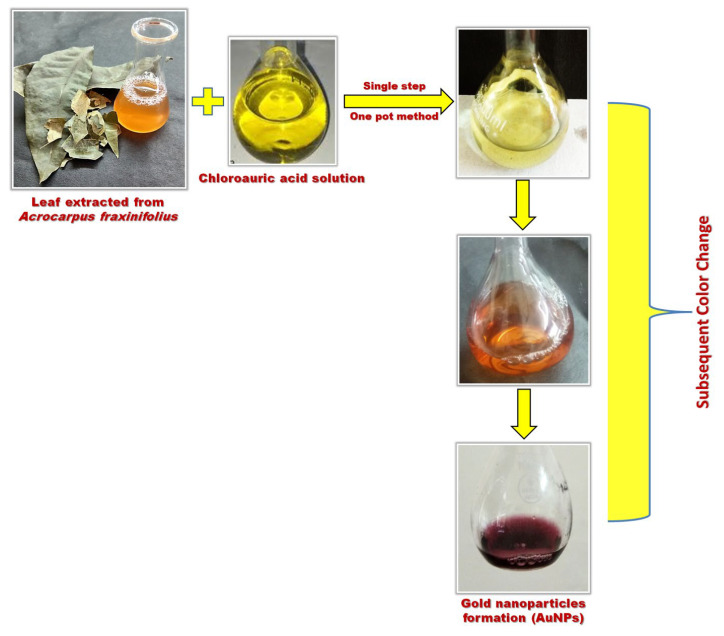
One pot green synthesis of gold nanoparticles (AuNPs).

Ten millimole concentration of the gold solution was prepared by mixing the chloroauric acid powder into distilled water. A 9 mL extract of *Acrocarpus fraxinifolius* was added to the gold solution and reserved in a dark place to start the reduction process. Biosynthesis or the reduction process began within a few minutes. The initial pale yellow color significantly changed to pink and eventually dark pink as the reduction proceeded (Scheme 2). The subsequent change in color is due to the reduction of Au^+^ to Au particles [41]. All the reactions were conducted at ambient temperature.

### 2.4. Synthesis of Hydroxyapatite (HA) Nanopowder

HA nanopowders were set up through the notable wet chemical precipitation method [29,42,43]. This involved adding 70.92 × 10^−3^ mol/liter of calcium oxide (CaO) and 13.8 × 10^−3^ mol/liter deionized water to a beaker and keeping it for stirring at 1000 rpm for 20 °C. After 24 h of reaction, it formed a suspension of Ca(OH)_2_ basic solution with pH 12.86. The temperature was maintained by a temperature probe. Then, 4.92 × 10^−3^ mol/liter orthophosphoric acid was added to the basic suspension at a rate of 1.5ml/min. pH was monitored after every addition of acid using a METTLER TOLEDO (Columbus, Ohio, United States) pH meter by maintaining a pH of 6.87 with an accuracy of ± 0.2. Again, it was kept for stirring to support the maturation at 1000 rpm at 20 °C. The ripening of the slurry that occurred after the maturation stage was done by using a 1.37 × 10^−3^ mol/liter ammonia solution. Synthesized material was dried in a hot air oven at the 100 °C for 1 h. After drying the HA powder, it was kept in a crucible for annealing at 600 °C temperature.

### 2.5. Preparation of Hydroxyapatite (HA)/Gold (Au) Nanocomposite

**Scheme 3:**
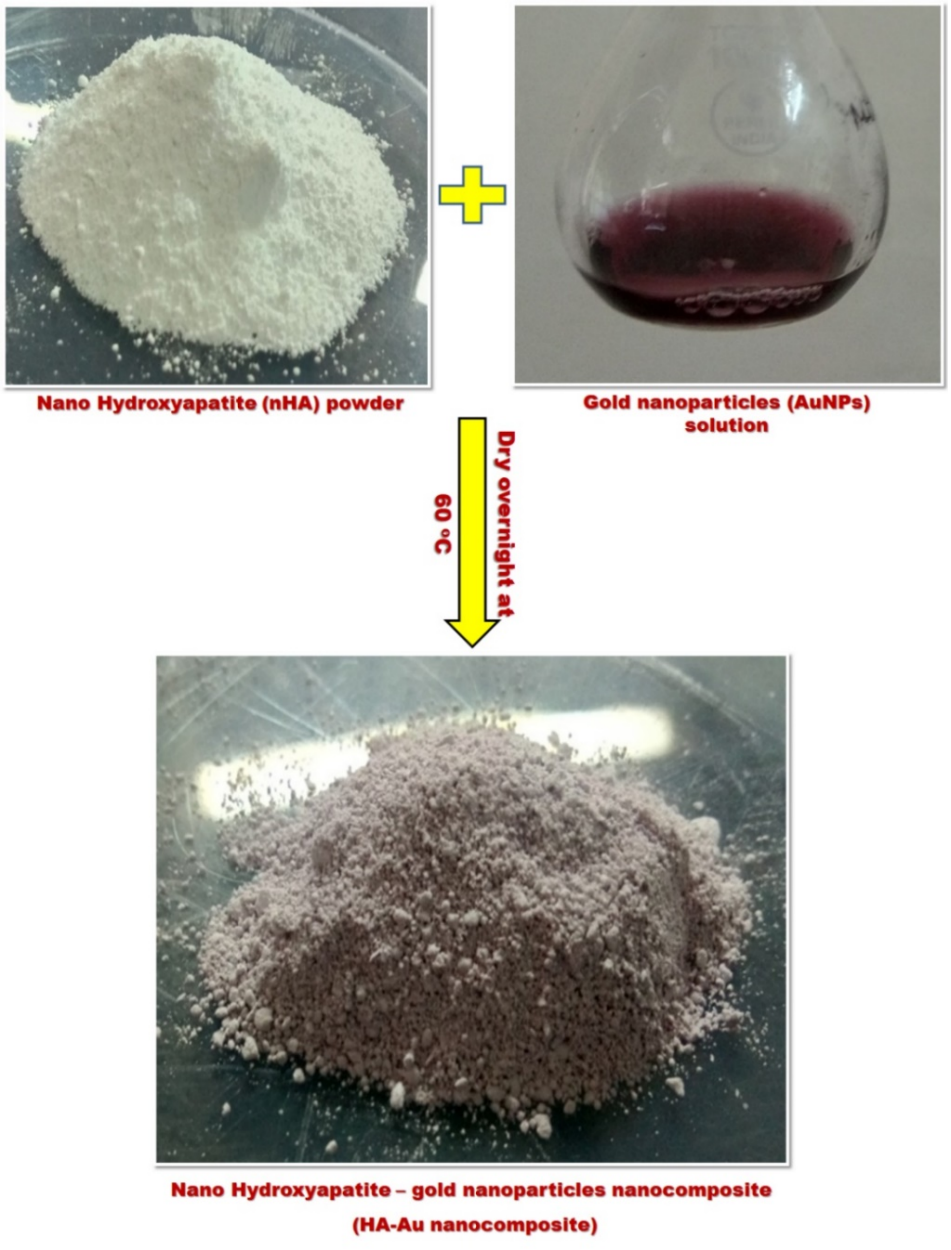
Preparation of HA-Au nanocomposite.

Nanocomposites were synthesized by the ex-situ method. Twenty milliliters of prepared AuNPs solution were taken and mixed properly with 1 gm of as-prepared HA nanopowder and were kept undisturbed for at least 2 h. After some time, it was kept for drying in a hot air oven at around 60 °C overnight (Scheme 3). After complete drying, the composite was scraped off and powdered using a mortar and pestle and was further used for investigations.

### 2.6. Physico-Chemical Characterization

Phase analyses were done by using a Spinner PW3064, X’pert PRO Diffractometer (United Kingdom) with Cu-Kα of 0.154056 nm at the 2θ scale. The average crystallite sizes (*D*) of the HA nanopowders were estimated from the obtained XRD data by employing the Scherrer formula [43]. Synthesized samples were characterized by an FTIR spectrophotometer (PerkinElmer, Inc., Waltham, MA, USA) equipped with ZnSe-diamond crystal as the focusing element. The surface morphology of the nanocomposites was studied by SEM (JSM-6490LV, JEOL, Ltd., Tokyo, Japan). Prior to the estimation, the samples were dried in air and sputter-coated with gold. UV-Visible spectroscopy analysis of gold nanoparticles and dye adsorption studies were performed using UV2550 (Shimadzu, Kyoto, Japan) spectrophotometer. The adsorption kinetics and isotherm protocols are provided in the Supplementary Materials.

### 2.7. Adsorption Studies

The batch adsorption experiments of methylene blue (MB) on HA/Au nanocomposite were studied to measure the effect of adsorbent dose, initial dye concentration, pH, and temperature using a UV-visible spectrophotometer [44]. A stock solution of MB (500 mgL^−1^) was prepared and further diluted to the desired concentration. A known weight of the adsorbent was taken in a 100 mL dye solution for each experiment and absorbance of residual dye solution was measured after every 10 min by a UV-visible spectrophotometer at a wavelength of 663 nm. The amount of dye absorbed per unit mass of composites (*qt*) was determined by applying Equation (1):

(1) $$q_{t}=V\;\frac{\left(C_{t}-C_{o}\right)}{W},\;$$

where *V* is the volume of dye solution (*L*), *W* is the mass of composite (*g*) *C_t_* and *C_o_* is the initial and final concentration at different time intervals.

### 2.8. Antibacterial Studies

The antibacterial activity of the nanocomposite samples was assayed against *Pseudomonas aeruginosa, Staphylococcus aureus and Micrococcus luteus* (obtained from IMTECH, Chandigarh) using the agar well diffusion method [45]. The complete details are provided in the Supplementary Materials.

## 3. Result and Discussion

### 3.1. Synthesis Mechanism

**Scheme 4:**
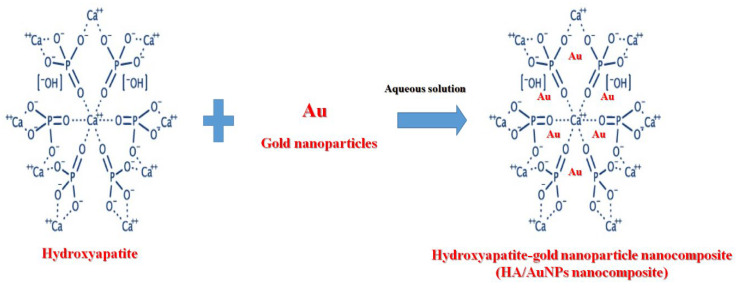
Schematic representation for the synthesis of HA/Au nanocomposite.

The primary goal of this study was to design HA/Au nanocomposite, bearing cationic as well as anionic functional moieties for the enhanced subtraction of MB dye molecules from the wastewater. To achieve the desired goal, HA powder was synthesized via a well-known wet precipitation method [43]. In the past few years, AuNPs have been employed in a large number of areas due to their chemical stability and exceptional properties [46,47,48,49,50]. To date, a variety of chemical and physical methods have been reported for the development of AuNPs; however, the utilization of poisonous chemicals and elevated temperature in the synthesis process, these strategies were seen as harmful and might be unsafe to the ecosystem [46,48,49]. The green synthesis of AuNPs is receiving attention because of the reduction of their salts at ambient temperature, environment-friendly, economical, and safe [46]. Aqueous leaf extract of *Acrocarpus fraxinifolius* as a novel source of phytochemical was used for the synthesis of AuNPs. It is accepted that the plant extract contains complex reducing moieties such as antioxidants, enzymes, and phenolic groups that reduce Au ions to Au particles through a phytochemical-driven reaction [46,48]. Therefore, no external stabilizing/capping agent is required during the synthesis process, since phytochemicals work at reducing as well as stabilizing agents [46]. The resultant solution is additionally incubated to reduce metal salt and outwardly checked by a color change (Scheme 2). The as-synthesized AuNPs suspension was added to the HA powder, where these AuNPs get caged inside the uniform pore geometry of the HA molecule [51] and resulted in the formation of a composite structure, as shown in Scheme 4.

### 3.2. Confirmation of Gold Nanoparticles (AuNPs) By Using UV-Vis Spectroscopy

**Figure 1:**
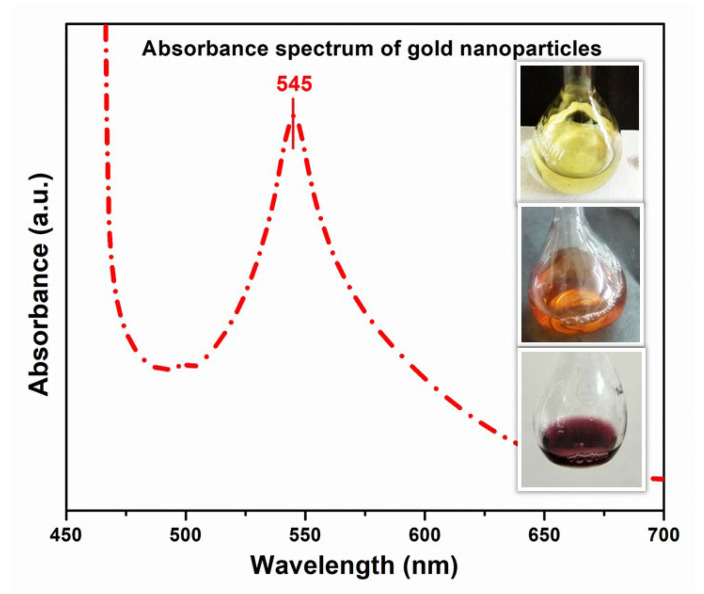
UV-Vis spectrum of the reaction mixture containing gold nanoparticles (AuNPs).

Many plant extracts have been used for the preparation of NPs and their use in a variety of applications [46,47,48,49,52]. Based on the literature survey, this is the first report for the use of *Acrocarpus fraxinifolius* plant extract for the biosynthesis of Au-NPs. The transformation of Au ions into Au atoms by using *Acrocarpus fraxinifolius* extract was observed through a change in color in the course of the reduction process and UV-vis spectroscopy. The colloidal suspension of Au particles shows a variation in the color change from pale yellow to purple because of the surface plasmon resonance (SPR) as displayed in the inset of Figure 1. Similar behavior is also described by different authors [47,48,49,52]. This change in color suggested the completion of the bioreduction process to transform Au ions into Au atoms [48]. The absorption spectra of Au-NPs exhibited a characteristic peak at around 545 nm due to the SPR of Au (Figure 1). Thus, the *Acrocarpus fraxinifolius* plant extract acted as a reducing and stabilizing agent during the biosynthesis of Au-NPs.

### 3.3. XRD Analysis

**Figure 2:**
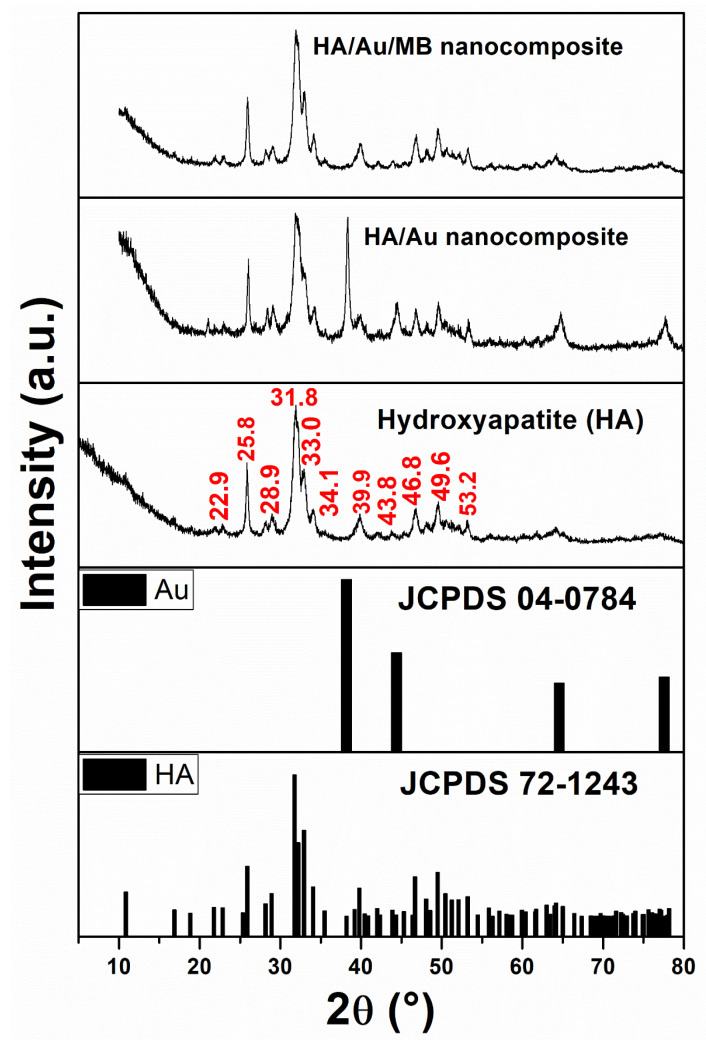
XRD patterns of the pure HA, HA/Au nanocomposite and HA/Au/MB nanocomposite.

The XRD patterns of as-prepared HA, HA/Au, and HA/Au/MB nanocomposites are displayed in Figure 2. The diffraction peaks were shown in the figure, which corresponds to different planes for HA. The diffraction peaks of HA were indexed to the hexagonal structure of HA with space group P63/m. The diffraction peaks of HA are in agreement with the JCPDS no. 72–1243 [42,53,54]. The peak at 2θ = 38.38° signifying Braggs reflection from (111) plane of Au and well-matched with the JCPDS file No. 04-0784 [55]. A clear shifting, as well as the formation of new peaks, were detected after the incorporation of AuNPs into the HA matrix. The variation in peak positions indicated the formation of nanocomposite based on AuNPs and HA. A similar variation is also observed in the case of the MB dye adsorbed in the HA/Au-NPs composite. The average crystallite size was found to be around 18.43 nm and 21.5 nm for HA and AuNPs, respectively.

### 3.4. FTIR Spectra Analysis

**Figure 3:**
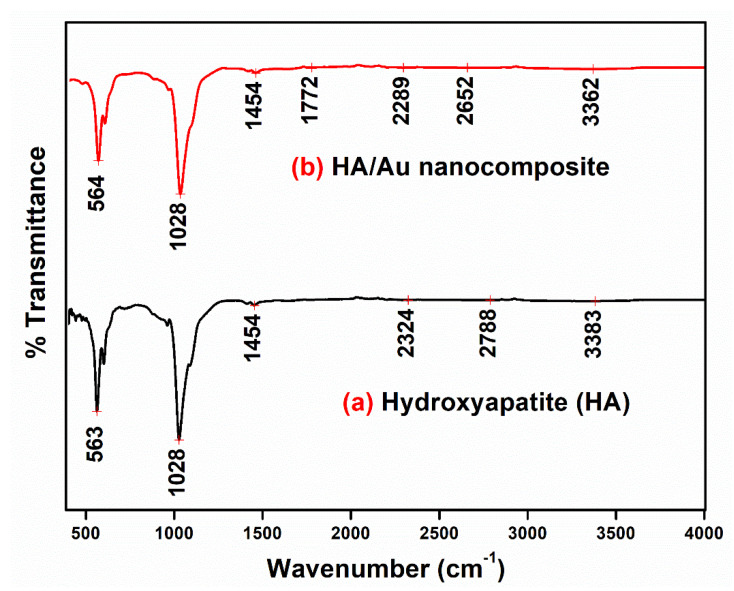
FTIR spectra of (**a**) pure HA and (**b**) HA/Au nanocomposite.

Figure 3 displays the FTIR spectra for HA and synthesized HA/Au nanocomposite, respectively. Here, the HA spectrum displays strong bands in the wavenumber region of 1040–560 cm^−1^, which are characteristics of the phosphate of the HA phase [42]. The sharp peak at 563 cm^−1^ and a small peak at around 509 cm^−1^ are ascribed to the bending mode of O–P–O [39,56,57]. The small band at 1454 cm^−1^ may be ascribed to the carboxylate group coordinated to the HA surface under different configurations [39,50]. A sharp peak is found at 1028 cm^−1^, which is attributed to the asymmetric stretching of phosphate ions (PO_4_^3–^) [29,34,43]. The HA/Au nanocomposite spectrum also shows all the peaks with a slight variation in the peak’s intensity and peak position. After impregnation of AuNPs, some bands were shifted due to the incorporation of AuNPs on the surface of HA, and some peaks were disappeared or having a reduction in intensity due to the trapping of AuNPs inside the HA matrix. However, the addition of AuNPs marks the absence of some peaks like 3383 and 2788 cm^−1^ which was initially presented in HA and the addition of a new peak at 1775 cm^−1^ which is due to COO– at the surface of –OH and C=O group in the production of Au-NPs [34,58]. The FTIR results clearly show the formation of HA and HA/Au nanocomposite.

### 3.5. SEM Results

**Figure 4:**
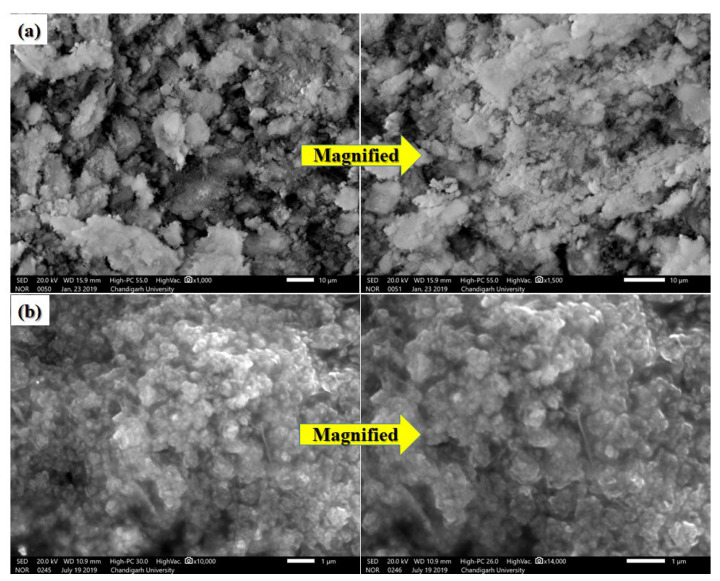
SEM images (**a**) HA and (**b**) HA/Au nanocomposite.

**Figure 5:**
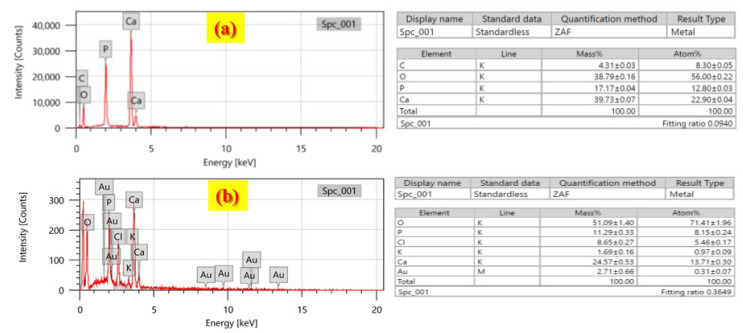
EDS spectra of (**a**) Hydroxyapatite and (**b**) HA/Au nanocomposite.

The morphologies of the HA and HA/Au nanocomposite are displayed in Figure 4a,b. The HA powder annealed at 600 °C showed fibrous particles with a porous structure [42]. The AuNPs incorporated HA showed very dense AuNPs on the surface of the HA. The elemental analysis of HA and HA/Au nanocomposite was done by using Energy-dispersive X-ray spectroscopy (EDX) to check the various elements present in the samples and to confirm the incorporation of AuNPs in the HA matrix (Figure 5a,b). The EDS curve (Figure 5a) portrays the existence of Ca and P in an extremely high amount, which can also be seen by the resultant histograms observed during EDX analysis. The presence of AuNPs on the surface of HA was confirmed by EDS (Figure 4b). The presence of Au peaks reflects that Au particles are incorporated into the HA matrix. The EDS spectra were taken in a complete area of square millimeter range to make sure the uniform distribution of Au-NPs.

### 3.6. Adsorption Studies

**Figure 6:**
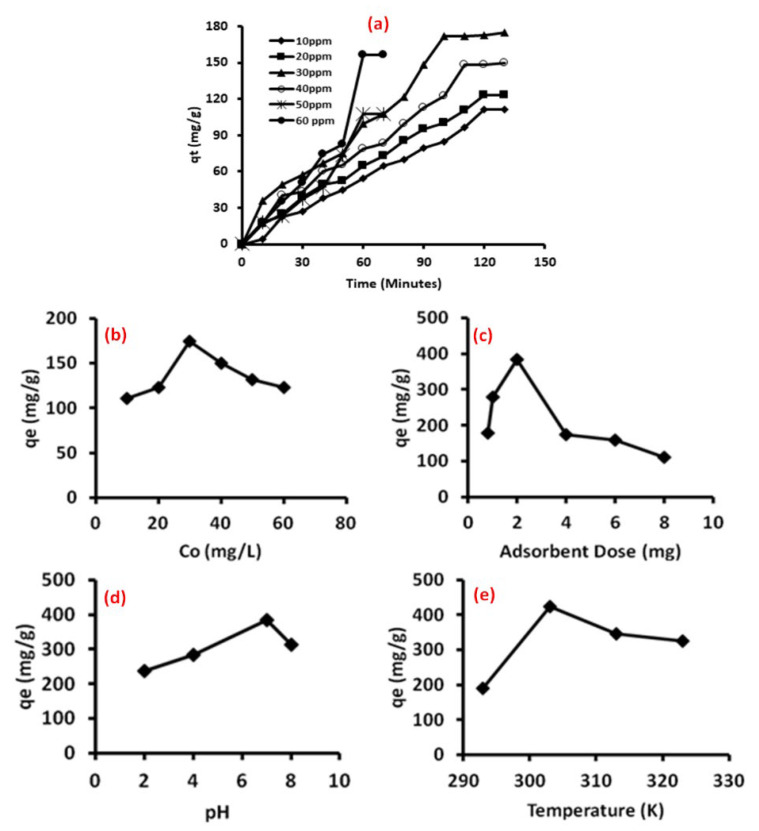
Effect of various parameters on adsorption capacity at equilibrium (**a**) Time, (**b**) concentration of dye, (**c**) adsorbent dose, (**d**) pH and (**e**) temperature.

The methylene blue (MB) dye adsorption on HA/Au nanocomposite was investigated by changing various parameters adsorption dose (0.8–8mg), adsorption time, pH (2.0–8.0), temperature (290–330 K) and dye concentration (10–60 ppm) (Figure 6). The effect of the initial concentration of dye was studied as the function of time of contact between dye and adsorbent; dye concentration was varied from 10 ppm to 60 ppm (Figure 6a,b). The adsorption capacity initially rises with a rise in dye concentration up to 30 ppm; afterward, it starts decreasing. Initially, the driving force for mass transfer increases with the increase in the dye concentration which results in more MB adsorption. Further increases in MB concentration result in an excess increase in dye molecules that causes a larger contact time between the active site over adsorbent and adsorbate that leads to saturation and hence adsorption capacity decreases [39,59,60]. At 30 ppm, equilibrium was attained at 90 min of contact time whereas at higher concentrations (50 ppm and 60 ppm) equilibrium is attained early but has low adsorption capacity.

The influence of the adsorbent dose is prominent over the adsorption capacity of HA/Au nanocomposite [6]. The amount of adsorbent varies from 0.8–10 mg (Figure 6c). The optimal adsorption capacity was observed at 2.0 mg adsorbent dose. The rise in the adsorbent dose from 0.8 mg to 2.0 mg leads to an increase in adsorption capacity. This is due to the accessibility of more surface area and adsorption sites on HA/Au nanocomposite [39]. With the further increase in adsorbent dose, the agglomeration of the particles takes place, which in turn affected the surface area, and as a result, the adsorbent active sites become saturated, causing low adsorption capacity at a high adsorbent dose composite [39,61,62].

The effect of pH on the elimination of MB dye by HA/Au nanocomposite was explored at an ambient temperature from pH 2 to 8 while keeping other parameters constant (Figure 6d). The nanocomposite shows the maximum MB dye adsorption at neutral pH 7, thereafter it starts decreasing [63]. Usually, the MB dye shows the positively charged ions when dissolved in water [64,65]. At a lower pH, the adsorption of MB dye was improved; which may be due to the existence of an acidic medium, the surface of the HA/Au nanocomposite may get a positive charge, which does not pull the positively charged MB dye in the solution [64,66]. As the solution pH increases, the adsorbent surface acquires the negative charge which increases the adsorption capacity of HA/Au nanocomposite as a result of the development of electrostatic interaction between the negatively charged HA/Au nanocomposite and the positively charged MB dye [67]. A fall in adsorption of MB dye on HA/Au nanocomposite is observed at a basic pH. This is due to the fact that, at an alkaline pH, repulsion happens between the HA/Au nanocomposite adsorbent and alkaline medium of the MB dye solution. Furthermore, at an alkaline pH, the MB dye in the solution also acquired a negative charge and, as a result, the maximum adsorption of MB dye on HA/Au nanocomposite was found to be at pH 7.0. Similar behavior is also observed by various authors [64,67,68].

The adsorption capacity of MB dye over adsorbent is also investigated by changing the temperature from 290 K to 330 K (Figure 6e). The results revealed that the rise in the temperature of dye solutions increases the adsorption due to more availability of active sites because of activation of the adsorbent surface at higher temperatures. A further increase in temperature causes excess acceleration and diffusion in active moieties causes hindrance between active sites and hence adsorption decreased [61].

### 3.7. Adsorption Kinetics

**Figure 7:**
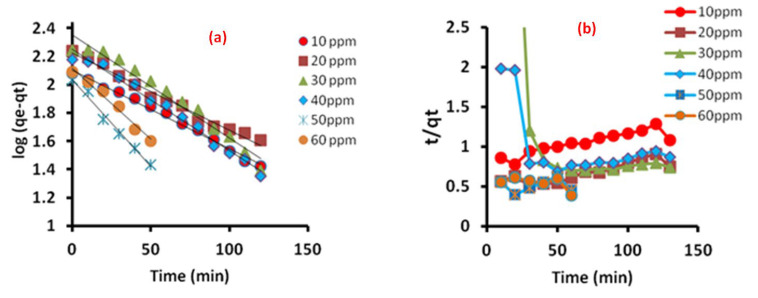
(**a**) Pseudo- first order and (**b**) pseudo-second order kinetic model for dye adsorption.

The adsorption mechanism of MB onto HA/Au nanocomposite was calculated with the help of Lagergren pseudo-first-order and pseudo-second-order kinetic model (Supplementary Materials). The pseudo-first-order and pseudo-second-order kinetic model for dye adsorption is presented in Figure 7. The plot of log (q_e_-q_t_) vs t (Pseudo-first order, Figure 7a) and t/q_t_ vs t (pseudo-second-order, Figure 7b) indicated that the sorption process obeyed pseudo-first-order kinetics, as theoretical values of q_e_ (Table 1) are closer to experimental values of q_e_ for the pseudo-first-order kinetic model, whereas, with q_e_ values calculated by pseudo-second-order, the kinetic model reveals large variance from the experimental value. In addition, the linear fitting of the pseudo-first-order model gives much higher values of correlation coefficient (R^2^) as compared to the pseudo-second-order kinetic model (Table 1). This shows that the pseudo-first-order model was a good fit for the experimental data, which indicates that the process involves physisorption, and that the dye molecules interact by forming electrostatic ion pairs with the functional groups of the adsorbent.

### 3.8. Validity of Kinetic Models

The kinetic models were also validated for their appropriateness by normalized standard deviation Δ*q*(%), which is given in Equation (2).

(2) $$\Delta q\left(\%\right)=100\times \sum^{}\frac{{\left[\left(q_{\exp\;}-q_{cal}\right)/q_{exp}\right]}^{2}}{N-1},$$

where *N* is the number of data points, *q_exp_* and *q_cal_* (mg/g), are the experimental and calculated adsorption capacity, respectively.

The smaller value of Δ*q*(%) designates a better fit of the model. It is evident from Table 1 that the value of Δ*q*(%) was found to be in the range of 1.41–6.72% for the first-order model, whereas the second-order model has a comparatively bigger value. This is in accordance with the R^2^ values attained for both models and confirm that the adsorption of the dye could be properly described by pseudo-first-order kinetic model.

### 3.9. Adsorption Isotherm

**Figure 8:**
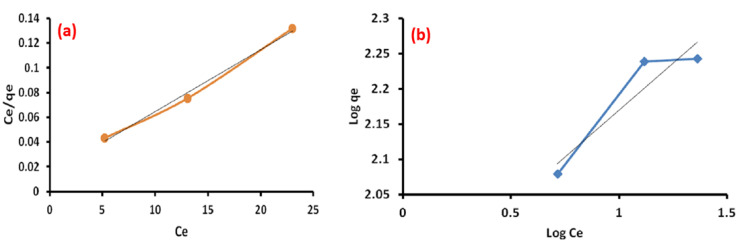
Plot of (**a**) Langmuir adsorption isotherm and (**b**) Freundlich adsorption isotherm.

The type of adsorption can be confirmed by validating the experimental data with the help of various adsorption isotherm models. The equilibrium adsorption isotherm can be shown by plotting the solid-phase concentration against the liquid phase concentration. In the present case, the Langmuir and Freundlich adsorption isotherm models (Supplementary Materials) are employed in the equilibrium data to examine the removal of dye using the HA/Au nanocomposite. As per the Langmuir adsorption isotherm model, adsorption arises on a homogenous adsorbent surface of equally available and energetically equivalent sites carrying equal numbers of molecules, and there is no interaction between adsorbate molecules once all the sites are occupied. All the sites are equivalent and there is no interaction of the adsorbed molecules with their neighboring molecules or site as the adsorption energy of a molecule is constant and does not rely on the active centers of an adsorbent. The Freundlich model gives non-ideal multi-layer sorption onto heterogeneous surfaces with a uniform energy distribution which has a reversible adsorption property. It shows that the dye concentration on the nanocomposite will keep on increasing as long as there is an addition of dye concentration to the solution as this is not confined to the monolayer of the adsorbent. The plot of ln qe vs. ln Ce; and Ce/qe vs. Ce (Figure 8) gave the values of Freundlich (K and *n*) and Langmuir (Q_m_ and b) constants and are given in Table 2.

Table 2 shows that Langmuir adsorption isotherm has a high value of correlation coefficient (R^2^ = 0.992) than Freundlich isotherm (R^2^ = 0.873), which indicates that the Langmuir model is more appropriate for studying sorption equilibrium. More suitability of the Langmuir model than the Freundlich model concludes that the adsorption of dye on the adsorbent surface is homogeneous. The maximum adsorption capacity reported in previous literature for hydroxyapatite-based materials for the removal of the dyes from aqueous media is presented in Table 3.

### 3.10. Thermodynamic Studies

The nature of adsorption was confirmed by evaluating thermodynamic parameters. The thermodynamic possibility and the spontaneous nature of the process can be determined by calculating thermodynamic constants, Gibbs free energy change (∆*G*^*O*^), enthalpy change (∆*H*^*O*^) and entropy change (∆*S*^*O*^). The von’t hoff equation was employed to determine the change in enthalpy (∆*H*^*O*^) and entropy (∆*S*^*O*^) as given below:

(3) $$\ln k_{c\;\;\;\;\;}=\frac{\Delta S^{O}}{R}-\frac{\Delta H^{O}}{RT}.$$

*K_c_* is the distribution coefficient. The *K_c_* value was calculated using Equation (4):

(4) $$k_{c}=\frac{q_{e}}{C_{e}},$$

where *qe* (mg/g) and *Ce* (mg/L) are the equilibrium adsorption and concentration of dye, respectively. T is the temperature in K and R is the gas constant [8.314 J/mol-K)]. Δ*H*^*O*^ and Δ*S*^*O*^ are measured from the slope and intercept of the plot between ln Kc and 1/T. Δ*G*^*O*^ was evaluated through Equation (5).

(5) $$\Delta G^{O\;}=-RT\ln k_{c}.$$

The values of Δ*G*^*O*^, Δ*H*^*O*^, and ∆*S*^*O*^ for the adsorption of dye onto HA/Au nanocomposite are given in Table 4. Negative values of Δ*G*^*O*^ reveal the spontaneous adsorption of MB by HA/Au nanocomposite [34]. Positive values of Δ*H*^*O*^, which is supported by enhanced adsorption at high temperatures, reflect that adsorption of MB by HA/Au nanocomposite exhibited an endothermic process of adsorption [34]. The positive value of Δ*S*^*O*^ and Δ*H*^*O*^ reflected the affinity for MB adsorption by HA/Au nanocomposite and possibly be managed by an entropy effect rather than enthalpy change. This type of behavior is also observed by various authors [34,39].

### 3.11. Mechanism of Dye Adsorption

**Scheme 5:**
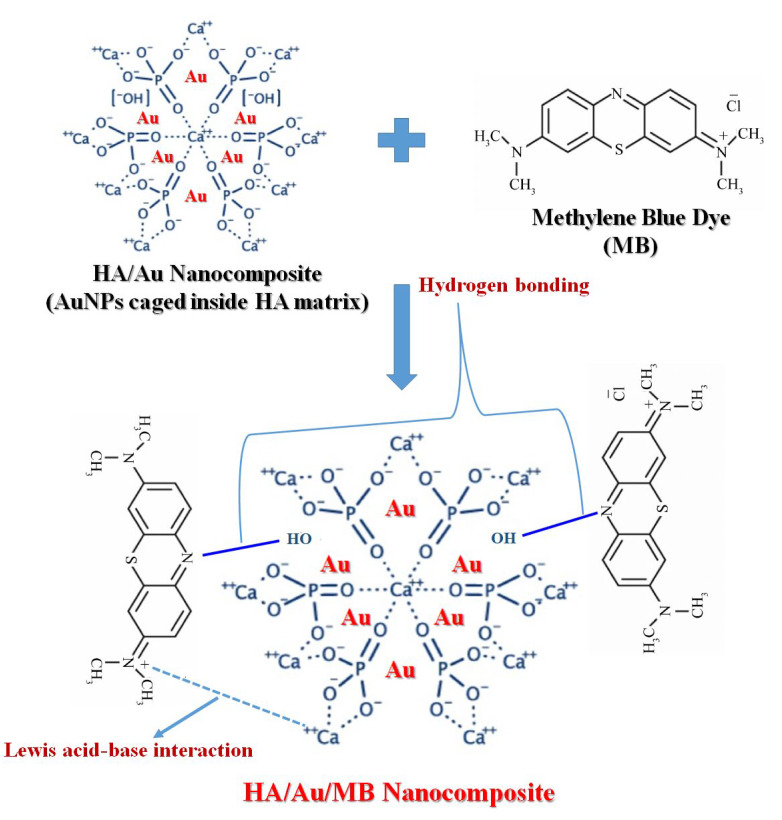
Schematic mechanism of adsorption of MB dye on to the HA/Au nanocomposite.

The efficient adsorption of MB on HA/Au nanocomposite may be initiated by many factors. The possible mechanism of dye adsorption is shown in Scheme 5. The fast adsorption rate of MB over HA/Au nanocomposite is possibly due to the nonexistence of internal diffusion resistance and a large number of vacant adsorption sites, which permit the rapid adsorption of MB from the aqueous solution [73,74]. HA/Au nanocomposite has AuNPs in its matrix structure and these AuNPs act as a donor as well as an acceptor of electrons, which helps in electron transmit and favors catalytic activity [75]. MB is typically a cationic dye and HA exhibits amphoteric nature. The amphoteric structure of HA provides a number of active sites for MB dye molecules for interaction that leads to MB dye adsorption [76]. The strong H-bonding interaction between the P-OH group of HA/Au nanocomposite and the nitrogen atom of the MB molecules may possibly contribute to the adsorption of MB dye molecules. The nitrogen atoms of MB dye molecules may also interact with Ca^2+^ groups of HA via Lewis acid-base interaction [77]. In an acidic medium, positively charged (CaOH^2+^) and neutral ≡P–OH sites become more positive and make a surface charge over HA/Au positive hence adsorption decreased. At a higher pH neutral –CaOH moieties and negatively charged PO^–^ species predominated, causing the HA surface to become negatively charged. The electrostatic attraction between the negatively charged surface of HA/Au and the positively charged group of the dye may have been enhanced and, as a result, adsorption takes place.

### 3.12. Antibacterial Activity Analysis

**Figure 9:**
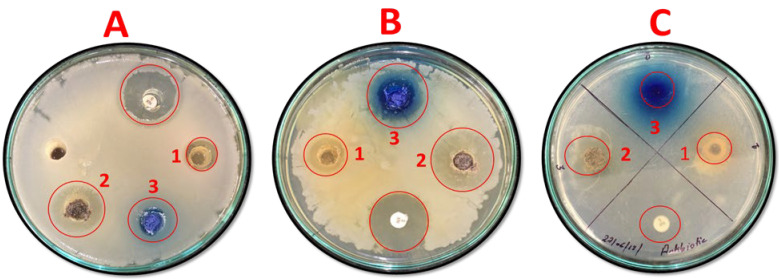
MHA plates having antibacterial activity of test sample 1(AuNPs), 2(HA/Au nanocomposite) and 3(dye adsorbed waste HA/Au nanocomposite) against bacterial strains, *M. luteus* (**A**) *S. aureus* (**B**) and *P. aeruginosa* (**C**).

The antibacterial ability of AuNPs (sample 1), HA/Au nanocomposite (sample 2), and dye adsorbed waste HA/Au nanocomposite (sample 3) samples were tested against *P. aeruginosa*, *M. Luteus* and *S. aureus* bacterial strains. *M. Luteus* is a Gram-positive bacteria and a constituent of the human buccal flora. *S. aureus* is also a Gram-positive, round-shaped bacterium that is a fellow of the Firmicutes. *P. aeruginosa* is an encapsulated, Gram-negative, rod-shaped bacterium and is responsible for disease in plants and animals, including humans [78]. For all the strains, AuNPs (sample 1) showed an average of 3mm zone of inhibition for all the bacteria (Figure 9 and Table 5). It is well reported that the AuNPs have more toxicity against many bacterial strains, but their ineffective binding surface interactions alone or without incorporation in any matrix reduced their possibility as an antibacterial agent against the infected bacterial surface [79]. It was observed that the HA/Au nanocomposite (sample 2) and dye adsorbed waste HA/Au nanocomposite (sample 3) showed more or less the same antibacterial activity against all the bacterial strains. Among all the samples the best antibacterial activity was observed against *S. aureus* for HA/Au nanocomposite, where a significant zone of inhibition (23 mm) was observed (Table 5). However, a 21 mm zone of inhibition was observed in the case of dye adsorbed waste HA/Au nanocomposite (sample 3). A 3 mm, 7 mm, and 5 mm zone of inhibition was observed against *M. luteus* for AuNPs (sample 1), HA/Au nanocomposite (sample 2) and dye adsorbed waste HA/Au nanocomposite (sample 3), respectively. On the other hand, a 2 mm, 4 mm and 3 mm zone of inhibition was observed against *P. aeruginosa* for AuNPs (sample 1), HA/Au nanocomposite (sample 2), and dye adsorbed waste HA/Au nanocomposite (sample 3), respectively. It was observed that the HA/Au and dye adsorbed HA/Au nanocomposites exhibited better antibacterial activity against Gram-positive bacteria as compared to the gram-negative bacteria. The gram-negative bacteria are more susceptible to antibodies and antibiotics than gram-positive bacteria, as they have a comparatively a bigger cell wall [78]. The cell walls of a gram-negative bacterium are composed of lipopolysaccharides that impart it with a negative charge Peptidoglycan and teichoic acid produce an overall positive charge over Gram-positive bacteria [79]. The antibacterial activity of gold nanocomposites is because of the diffusion of AuNPs inside bacterial cell membranes, causing a blockage in the functions of respiratory chain proteins and transport proteins leads to faded respiration and permeability [80]. The AuNPs cause the breakage of Cys-Cys sulfur bridges of protein chains and interfere with the functioning of cytoplasm, nucleic acid and DNA [81,82]. The diffusion of AuNPs inside the cell membrane of gram-positive and gram-negative bacteria also causes hindrance in cell division. The contact of AuNPs with dissolved oxygen produces excessive reactive oxygen species that lead to oxidative stress and, as a result, help in the free radical attacks onto the membrane lipids. This leads to the death of the bacteria by the collapse of the cell membrane [83].

## 4. Conclusions

The preparation of hydroxyapatite nanopowder was successfully carried out by a wet chemical precipitation method, followed by the impregnation of AuNPs into the hydroxyapatite nanopowder matrix. The results of the XRD analysis confirmed the formation of hydroxyapatite nanopowder and AuNPs. The experimental data fit well with the pseudo-first-order kinetic model as the values of the correlation coefficient are close to unity. The HA/Au nanocomposite would be used as a suitable adsorbent for the removal of dyes from wastewater. The output of thermodynamic modeling showed that the adsorption process was spontaneous and endothermic, and the positive value of Δ*S^o^* suggested the affinity of dye molecules to the HA/Au nanocomposite surface. The secondary waste generated from the adsorption of MB dye onto HA/Au nanocomposite has been applied effectively in the antibacterial application. The use of dye adsorbed waste reduces the secondary waste produced and is beneficial from a cost point of view. The results illustrated that the HA/Au nanocomposite and dye adsorbed waste HA/Au nanocomposite exhibited about the same antibacterial activity against *S. aureus*. Hence, the synthesized nanocomposite can be used as a potential material in MB dye adsorption and as an effective antibacterial agent for medical applications.

## Figures and Tables

**Table 1:** Comparison of pseudo first order and pseudo second order for the adsorption of MB dye on adsorbent at different initial concentrations of dye.

Conc. (ppm)	Pseudo First Order Kinetics					Pseudo Second Order Kinetics			
	q_e_(exp) (mg/g)	k_1_ (min^−1^)	q_e_(cal) (mg/g)	R^2^	Δ*q* (%)	k_2_ (g mg ^−1^ min ^−1^)	q_e_(cal) (mg/g)	R^2^	Δ*q* (%)
10	120	1.28 × 10^−2^	127.2	0.991	1.27	117871.2	312.5	0.7926	15.0
20	173.2	0.85 × 10^−2^	171.1	0.981	1.41	1026259	909.0	0.1171	18.5
30	174.7	1.68 × 10^−2^	190.6	0.967	2.79	35.31249	−15.528	0.3179	12.4
40	149.8	1.66 × 10^−2^	157.7	0.984	4.14	19655.94	−166.6	0.2816	16.9
50	122.6	2.34 × 10^−2^	131.6	0.976	6.72	843870.5	−714.28	0.1513	19.3

**Table 2:** Comparison of Langmuir and Freundlich isotherm constants.

Langmuir Isotherm		Freundlich Isotherm	
Q_m_ (mg/g)	200	K_f_	79.9
b (L/mg)	0.337	n	3.75
R^2^	0.992	R^2^	0.871

**Table 3:** Comparison of various adsorbents with hydroxyapatite for the removal of the dyes from aqueous media.

Adsorbent	Adsorption Capacity (Q_max_) (mg/g)	Reference
Poultry eggshell derived apatite	127.9	[39]
Carboxymethyl cellulose/acrylamide/nano-hydroxyapatite composite hydrogels	29.52	[29]
Nano Hydroxyapatite/Chitosan Composite	30.2	[65]
Hydroxyapatite nano-rods	337.330	[40]
Porous hydroxyapatite (HAp) granules	6.32	[69]
3-mercaptopropyl trimethoxysilane-modified hydroxyapatite	625	[70]
Hydroxyapatite–chitosan–Montmorillonite thin film	243.18	[71]
Hydroxyapatite	50.25	[72]
HA/Au nanocomposite	200	Present work

**Table 4:** Thermodynamic parameters for MB dye adsorption on HA/Au nanocomposite surface.

Temp (K)	Thermodynamic Parameters		
	∆*G*^*O*^ (KJ/mol)	∆*H*^*O*^ (KJ/mol)	∆*S*^*O*^ (J/molK)
293	−6.05	33.3	134.37
303	−7.50		
313	−8.73		

**Table 5:** Zone of inhibition of test sample 1(AuNPs), 2(HA/Au nanocomposite) and 3(Dye adsorbed waste HA/Au nanocomposite) against *Micrococcus luteus, Staphylococcus aureus* and *Pseudomonas aeruginosa* strains.

Sample Code	Sample Name	Zone of Inhibition (mm)		
		* Microc occus luteus (A) *	* Staphylococcus aureus (B) *	* Pseudomonas aeruginosa * *(C)*
1	* AuNPs *	3	4	2
2	HA/Au nanocomposite	7	23	4
3	dye adsorbed waste HA/Au nanocomposite	5	21	3

## Data Availability

All data generated or analysed during this study are included in this published article (and its Supplementary Materials files). The datasets used and/or analysed during the current study are available from the corresponding author on reasonable request.

## Supplementary Materials

The following are available online at https://www.mdpi.com/article/10.3390/nano11061403/s1.
