# Supplementary material for: Methylene Blue Dye Adsorption from Wastewater Using Hydroxyapatite/Gold Nanocomposite: Kinetic and Thermodynamics Studies
Source: Nanomaterials (Basel). 2021 May 26;11(6):1403. doi: 10.3390/nano11061403 (PMC8227305; doi:10.3390/nano11061403)
Supplement: Supplementary file 1 [file nanomaterials-11-01403-s001.zip › nanomaterials-1201656-supplementary/nanomaterials-1201656-supplementary.pdf]

nanomaterials

# Methylene Blue Dye Adsorption from Wastewater Using Hydroxyapatite/Gold Nanoparticles Composites: Kinetic and Thermodynamics Studies

**Kashma Sharma [1,2], Shreya Sharma [1], Vipasha Sharma [3], Pawan Kumar Mishra [4], Adam Ekielski [5], Vishal Sharma [1,*] and Vijay Kumar [6,*]**

[1] Institute of Forensic Science & Criminology, Panjab University, Chandigarh 160014, India; shama2788@gmail.com (K.S.); sharmashreya.m1223@gmail.com (S.S.)
[2] Department of Chemistry, DAV College, Sector-10, Chandigarh 160011, India
[3] Department of Biotechnology, Chandigarh University, Gharuan, Punjab 140413, India; sharma.vipasha@gmail.com
[4] Faculty of Business and Economics, Mendel University in Brno, 61300 Brno, Czech Republic; xmishra2@gmail.com
[5] Department of Production Engineering, Warsaw University of Life Sciences, 02-776 Warsaw, Poland; adam_ekielski@sggw.edu.pl
[6] Department of Physics, National Institute of Technology Srinagar-190006, Jammu and Kashmir, India
* Correspondence: vsharma@pu.ac.in (V.S.); vj.physics@gmail.com (V.K.)

## Supplementary information

### Adsorption Kinetics

Lagergren pseudo-first-order equation shows that the rate of change of solute uptake with time is directly associated to the variation in the quantity of solute uptake at equilibrium and the volume of solute uptake with time is given by Eq (1):

$$\frac{dq_t}{dt} = k_1 (q_e - q_t), \tag{1}$$

On integrating equation (1) within the limits $q_t$=0 at t=0 to $q_t$=$q_e$ at t=t and converting it into linear form, the equation become

$$log\ (q_e - q_t) = log q_e - \frac{k_1}{2.303} t, \tag{2}$$

where $q_e$ and $q_t$ are the adsorption capacity at equilibrium and at time t (min), respectively. $k_1$ is the rate constant of pseudo-first-order adsorption ($min^{-1}$). The values of $k_1$ and $q_e$ can be estimated from the graph of log ($q_e$-$q_t$) vs t.

Pseudo-second-order equation is written as:

$$\frac{dq_t}{dt} = k_2 (q_e - q_t)^2, \tag{3}$$

On integrating the above equation within the limits and changing into linear form, we got an equation (4):

$$\frac{t}{q_t} = \frac{1}{k_2 q_e^2} + \frac{1}{q_e} t, \tag{4}$$

Where $k_2$ is pseudo-second-order rate constant (g $mg^{-1}min^{-1}$). The values of $k_1$, $k_2$, qe and regression coefficients are listed in Table 1 for different concentrations ranging from 10-60 ppm.

**Adsorption Isotherm**

The linear form of Langmuir and Freundlich is represented in Eqs. (5) and (6), respectively.

$$\frac{C_e}{q_e} = \frac{1}{Q_m b} + \frac{1}{Q_m} C_e \quad , \tag{5}$$

$$ln\, q_e = lnK + (1/n)\, ln\, C_e, \tag{6}$$

Where, $Q_m$ (mg/g) and b (L/mg) are Langmuir constants representing monolayer adsorption capacity and heat of adsorption respectively. K (mg/g) and n are Freundlich constants denoting adsorption capacity and adsorption intensity respectively. qe is the adsorption capacity (mg/g) of dye and Ce is the equilibrium concentration (mg/L) of dye solution.

**Antibacterial studies**

The antibacterial activities of the AuNPs solution, HA/Au nanocomposite and dye adsorbed waste HA/Au nanocomposite samples were studied by agar well diffusion assay according the standard rules and recently reported procedure (Sharma et al. 2020). The overnight bacterial culture was diluted (1:100) to get $10^5$ CFU/ml for each bacteria to be studied (*Pseudomonas aeruginosa, Staphylococcus aureus and Micrococcus luteus*) and was dispersed employing sterile swab comprising a bacterial suspension on Muller Hinton Agar plates. Thereafter, wells having sizes of 6 mm diameter were punched into the agar plates involving a bacterial suspension and loaded with 50 mg/ml of AuNPs and synthesized nanocomposite samples and held at room temperature for 2 hours for dispersion. The plates were then incubated at 37 °C for 24 hours. The diameters around the inhibition zones were measured in mm after the incubation of 24 h against all the bacterial strains.
